# Immersion anaesthesia with ethanol in African giant land snails (*Acathina fulica*)

**DOI:** 10.1016/j.heliyon.2019.e01546

**Published:** 2019-04-25

**Authors:** Dario d’Ovidio, Paolo Monticelli, Mario Santoro, Chiara Adami

**Affiliations:** a Private Practitioner, Via C. Colombo 118, 80022, Arzano, NA, Italy; b Dep. of Clinical Sciences and Services, Royal Veterinary College, University of London, Hawkshead Campus, AL97TA, Hatfield, UK; c Istituto Zooprofilattico Sperimentale del Mezzogiorno, Via Salute 2, 80055, Portici, NA, Italy

**Keywords:** Neuroscience, Toxicology

## Abstract

Giant African land snails (*Achatina fulica*) are becoming increasingly popular pets and may be anaesthetised to allow diagnostics and surgical procedures. The objective of the present study was to evaluate the anaesthetic effects and anaesthetic-related complications of immersion in 5% ethanol in client-owned African pet land snails, anaesthetised to allow biopsies of the foot for screening of parasites. Variables such as minutes elapsing from immersion to anaesthetic induction and from removal from the bath to return of tentacle withdrawal reflex and recovery from anaesthesia were recorded, as well as the occurrence of adverse effects. Of the 30 snails enrolled, one (3.3%) had a fatal outcome whereas the remaining 29 (96.7%) snails completed the study and recovered from anaesthesia. Time to anaesthetic induction was 25 [25–29] minutes. Recovery was prolonged in one snail, which required 210 minutes to regain normal muscular strength. Time from removal from the ethanol solution to return of tentacle withdrawal reflex was 20 [14–42] minutes. Beside death, other observed adverse effects were production of bubbles (n = 4; 13.3%), and mucus secretion (n = 4; 13.3%). Immersion in 5% ethanol may be regarded as suitable anaesthetic technique for African giant snails for brief and moderately invasive surgical procedures. Nevertheless, recovery from anaesthesia may be prolonged and unpredictable.

## 1 Introduction

There is an increasing worldwide demand for unconventional pets, including invertebrates. Among these, the giant African land snail (*Achatina fulica*) has gained increasing popularity within the last few years. Besides being kept as pets, these molluscs are often kept in zoos, or as part of private collections and conservation projects. A common indication for snail anaesthesia is the need to perform clinical (e.g. physical examination), surgical (e.g. shell fracture fixation) or diagnostic (e.g. biopsies) procedures [1]. Unfortunately, very little is published about the anaesthetic management of these gastropods, particularly those kept as pets and, to the best of the authors' knowledge, there are no prospective studies focusing on the anaesthetic of *Achatina fulica*.

Immersion anaesthesia is a common method to anaesthetise various unconventional small-sized species, including amphibians and land snails, and various agents with anaesthetic properties, such as etomidate, alfaxalone and ethanol, have been used to prepare the anaesthetic bath solution [2, 3, 4, 5, 6]. One study investigated safety and efficacy of immersion anaesthesia, with various agents, in *Biomphalaria* snails and found that, whilst sodium thiopental was toxic to the snails and the association of Cetamine base with Tiazine chloridrate produced only partial anaesthetic effects, sodium pentobarbital resulted in safe and predictable anaesthesia [7]. Tricaine, also called MS222, has been used for immersion anaesthesia in various species of pulmonate snails, including *Biomphalaria, Helisoma, Bulinus* and *Lymnaea* [8]; similarly, menthol, either alone or in combination with chlorohydrate, has been used for bath immersion of *Lymnaea, Physa* and *Bulinus* snail species [9, 10].

The immersion in ethanol per se is not a novel technique; previous work suggests that 5% ethanol solution is an effective method to provide anaesthesia in land snails; however, the authors only reported that the snails recovered from anaesthesia within two hours from the end of immersion, and did not provide any detail pertaining to quality of recovery and occurrence of post-anaesthetic adverse effects [6].

The purpose of this prospective clinical trial was to evaluate the anaesthetic effects and anaesthetic-related complications of immersion in 5% ethanol, in 30 client-owned African pet land snails.

## 2 Materials and methods

An ethical approval was obtained from the Clinical Research Ethical Review Board of the Royal Veterinary College (University of London) prior to commencing the trial (license number: URN 2018-1804-3). The snails were presented at a referral practice for exotic animal species for diagnostics. Information about the general health of the animals were obtained through detailed anamnesis and visual exam, to exclude external damage or other lesions. Moreover, the muscle tone (ability to remain attached to the anaesthesist hand) and the tentacle withdrawal reflex (in response to pricking of the foot) were assessed preoperatively [1, 11]. General anaesthesia was required to perform biopsies from the foot muscle of the snails and process the specimens for screening of parasites (*Angiostrongylus cantonensis* and other nematodes), upon request of the owner.

The snails were transferred to the anaesthetic solution, prepared with 120 mL of dechlorinated water, by hand, always by the same operator wearing latex-free gloves. Water temperature was 22 ± 2 °C. Time of immersion, as well as the variables defined below, were recorded. Time to anaesthetic induction was defined as the minutes elapsed from the beginning of immersion in 5% ethanol and the achievement of anaesthetic induction, characterised by immobility and loss of tentacle withdrawal response to gentle stimulation [1, 11]. Time to recovery from anaesthesia was defined as the minutes elapsed from removal of the snails from the ethanol solution to regain of normal posture, muscular tone (defined as the ability to attach to the anaesthetist's hand) and tentacle withdrawal reflex in response to foot pricking with blunt forceps. The time elapsed from removal from the anaesthetic bath to return of tentacle withdrawal reflex was also annotated on the anaesthetic record. The occurrence of undesired effects of 5% ethanol, namely the production of bubbles, body retraction, expulsion of mucus and/or faeces, prolonged recovery (>2 h from removal from the anaesthetic bath), dehydration/desiccation, and death, were recorded.

Descriptive statistics applied, with the Kolmogorov Smirnov test used to analyse data distribution. A commercially available software was used for statistics (SigmaPlot 10 and SigmaStat 3.5, SYSTAT Software Inc, CA, USA).

## 3 Results

Data were not normally distributed and are presented as medians and 25–75% ranges. The shell of the snails, aged 1.6 [0.2–2] years, reached up to 7 inches in length. Of the 30 African snails included in the study, one (3.3%) had a fatal outcome approximately 20 minutes from immersion in the ethanol solution. The remaining 29 snails completed the study and recovered from anaesthesia and none of them showed any kind of reaction during surgical biopsy. Time to anaesthetic induction and time to recovery from anaesthesia were 25 [25–29] and 50 [31–75] minutes, respectively. Recovery was prolonged in one snail, which required 210 minutes to regain normal muscular strength. Time from removal from the ethanol solution to return of tentacle withdrawal reflex was 20 [14–42] minutes. Beside death, other observed adverse effects were production of bubbles in 4 out of 30 animals (13.3%) and mucus secretion in other 4 snails (13.3%); this accounted for a total proportion of snails showing adverse effects equal to 26.6% (n = 8/30). Within the 4 weeks following anaesthesia, the owner of the snails did not notice any change in behaviour or physical appearance in any of the animals.

## 4 Discussion

The results of this report suggest that immersion in 5% ethanol solution may be regarded as a suitable anaesthetic technique for African giant snails, as it consistently produces induction of general anaesthesia within a reasonable time. Nevertheless, the considerable variation in recovery time among snails, together with the observation of one prolonged recovery which lasted more than three hours from removal of the snail from the anaesthetic bath, raises the concern that recovery from anaesthesia of African giant snails after ethanol immersion may be prolonged and unpredictable.

Beside the one death that occurred during immersion, anaesthesia-related side effects were regarded by the authors as mild and were mostly represented by secretion of foamy mucus and bubbles. These adverse effects may be either the result of environmental stress or, alternatively, they may represent the attempt of the body to eliminate the anaesthetic agents, perceived as toxic substances [12]. Although the long-term follow up was limited to collection of information from the owner of the snails, it seemed that mucus and bubbles secretion was short term and did not result in long-term complications. Regarding the snail that died during immersion, although it is challenging to speculate about the causes of its death, it is hypothesised that hypoxia could have contributed to this adverse outcome. Similarly, hypoxia might have played a role also in the one prolonged recovery observed in another study snail.

Regarding the duration of surgical depth of anaesthesia, although this study was not designed to investigate the analgesic properties of ethanol, the relatively quick return of tentacle withdrawal reflex seems to indicate that, whilst full recovery might be prolonged, surgical anaesthesia may instead have short duration. If this was true, immersion in 5% ethanol would be suitable only for brief surgical procedures implying a mild to moderate nociceptive stimulation.

In conclusion, immersion in 5% ethanol produced reliable and consistent anaesthesia in African giant snails of duration sufficient to allow foot muscle surgical biopsies. The potential for side effects, together with the lack of evidence of effective and long-lasting antinociception, seems to suggest that the use of this anaesthetic technique should be limited to healthy snails undergoing non-invasive or minimally invasive short clinical procedures.

## Declarations

### Author contribution statement

Dario d'Ovidio: Conceived and designed the experiments; Performed the experiments; Contributed reagents, materials, analysis tools or data; Wrote the paper.

Paolo Monticelli: Conceived and designed the experiments.

Mario Santoro: Contributed reagents, materials, analysis tools or data.

Chiara Adami: Conceived and designed the experiments; Analyzed and interpreted the data; Wrote the paper.

### Funding statement

This research did not receive any specific grant from funding agencies in the public, commercial, or not-for-profit sectors.

### Competing interest statement

The authors declare no conflict of interest.

### Additional information

No additional information is available for this paper.
